# Como Devemos Investigar a Lesão Cardiovascular em Pacientes Pediátricos com COVID-19 Criticamente Enfermos em um Cenário de Vulnerabilidade Socioeconômica?

**DOI:** 10.36660/abc.20220254

**Published:** 2022-05-04

**Authors:** Gabriela Nunes Leal

**Affiliations:** 1 Universidade de São Paulo Hospital das Clínicas Faculdade de Medicina Universidade de São Paulo São Paulo SP Brasil Instituto da Criança e do Adolescente do Hospital das Clínicas da Faculdade de Medicina da Universidade de São Paulo, São Paulo, SP - Brasil; 2 Hospital Sírio Libanês Hospital Sírio Libanês São Paulo SP Brasil Hospital Sírio Libanês, São Paulo, SP - Brasil; 3 Hospital do Coração Hospital do Coração São Paulo SP Brasil Hospital do Coração, São Paulo, SP - Brasil; 4 Hospital e Maternidade São Luiz Itaim São Paulo SP Brasil Hospital e Maternidade São Luiz Itaim, São Paulo, SP - Brasil

**Keywords:** Crianças, COVID-19/complicações, Doenças Cardiovasculares/complicações, Síndrome Respiratória Aguda Grave, Inflamação, Hospitalização

**Figura 1:**
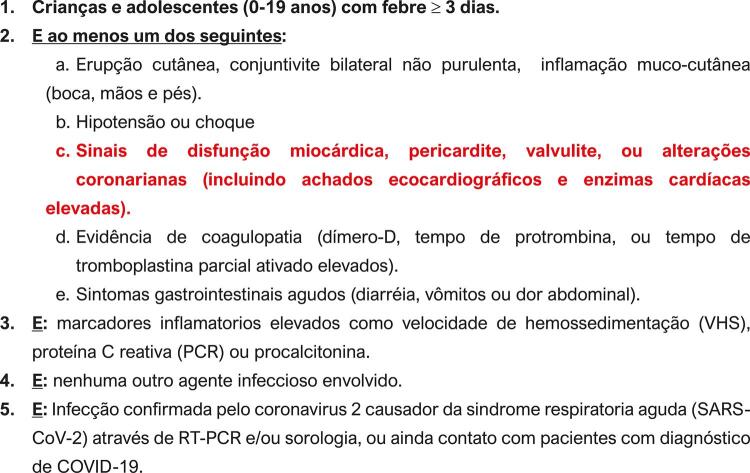
– Critérios de definição da Organização Mundial da Saúde (OMS) para síndrome inflamatória multissistêmica em pediatria (SIM-P), associada à COVID-19.

A literatura publicada em todo mundo documentou extensivamente a injúria cardiovascular em pacientes críticos com COVID-19. O envolvimento cardíaco parece ser uma característica proeminente da doença entre os adultos, ocorrendo em 20% a 30% dos pacientes hospitalizados e contribuíndo para 40% das mortes.^1^ Crianças e adolescentes são em geral poupados da COVID-19, com poucos apresentando sintomas. No entanto, a descrição da Síndrome Inflamatória Multissistêmica em Pediatria (SIM-P) reforçou que, apesar de raras, a apresentação clínica grave e a morte são possíveis na população pediátrica.^2^ O comprometimento cardiovascular na SIM-P associado à COVID-19 é frequente, constituindo inclusive um dos critérios diagnósticos para essa condição patológica segundo da Organização Mundial da Saúde (OMS) ( Figura 1 ).^3 , 4^

Feldstein et al. detectaram 80% de comprometimento cardíaco em um grupo de 186 pacientes com SIM-P, de 26 estados americanos. Cabe notar que 91% desses pacientes haviam realizado pelo menos um ecocardiograma durante a internação hospitalar.^5^ Dados nacionais recentes documentaram 48% de anormalidades ecocardiográficas em uma coorte de pacientes pediátricos hospitalizados por COVID-19 em um único centro, associadas à presença de SIM-P, admissão à unidade de terapia intensiva pediátrica, disfunção de múltiplos órgãos, necessidade de suporte ventilatório/inotrópico e morte. No mesmo estudo, a disfunção sistólica ventricular e os aneurismas coronarianos detectados ao ecocardiograma foram associados a níveis mais altos de troponina, assim como dímero-D e marcadores inflamatórios.^6^

Taxas de mortalidade mais altas devido à COVID-19 em crianças foram identificadas no Brasil, em relação aos demais países (8,2% x 1%), principalmente em decorrência de vulnerabilidade social a acesso limitado a suporte médico adequado. Uma vez que frequentemente faltam dados sobre a mortalidade de pacientes que não foram internados, é esperado que o efeito da pandemia sobre a população pediátrica seja ainda subestimado.^7^

De acordo com dados robustos coletados de 5857 pacientes com menos de 20 anos, todos hospitalizados por COVID-19 confirmada laboratorialmente, condições socioeconômicas, regionais e étnicas parecem moldar a mortalidade de crianças com COVID-19 no Brasil. Quando comparadas a crianças brancas, indígenas e pardas apresentam maior chance de morrer (OR: 5,83, IC 95%: 2,43 a 14,02; OR: 1,93, IC 95%: 1,48 a 2,51 respectivamente). Os autores também encontraram influência regional (maior taxa de mortalidade no norte – OR: 3,4, IC 95%: 2,48 a 4,65) e associação com com a condição socioeconômica (mortalidade menor entre crianças originárias de municípios mais desenvolvidos – OR: 0,26, IC 95%: 0,17 a 0,38).^8^

Complicações cardiovasculares que trazem risco à vida podem não ser prontamente reconhecidas em cenários de poucos recursos, contribuíndo para desfechos desfavoráveis em pacientes pediátricos críticos com COVID-19. Ferramentas de imagem de alto custo, como tomografia computadorizada ou ressonância magnética, estão geralmente indisponíveis. Mesmo um ecocardiograma à beira do leito, sugerido pela maioria dos guias de manejo da SIM-P, pode não estar acessível à admissão.^9^

A identificação de parâmetros laboratoriais em apresentação precoce, que possam levantar suspeita de comprometimento cardiovascular e a necessidade de cuidado intensivo é crucial, particularmente na ausência de recursos de imagem.

Um elegante estudo multinacional envolvendo jovens latinoamericanos examinou características próprias da síndrome respiratória aguda grave secundária à COVID-19 e da SIM-P, com e sem comprometimento cardiovascular. Foram incluídos 98 pacientes de 32 centros, distribuídos em 10 países da América Central, América do Sul e México. O grupo com comprometimento cardiovascular foi definido pelo diagnóstico de: taquicardia atrial ou ventricular, sustentada ou não; bloqueio atrioventricular de qualquer grau; dilatação de qualquer segmento coronariano (escore-z > +2); fração de ejeção de ventrículo esquerdo abaixo de 50%; dilatação ventricular esquerda (escore-z do diâmetro diastólico > +2); regurgitação moderada/ importante de valvas atrioventriculares ou semilunares; derrame pericárdico; miocardite pelo médico responsável; edema periférico; trombose/embolia vascular.^9^

48 pacientes apresentaram comprometimento cardiovascular e 50 não. O gupo com comprometimento cardiovascular teve maior frequência de admissão à unidade de terapia intensiva (77% vs 54%, p = 0,02); ventilação invasiva (23% vs 4%, p = 0,007) e de suporte inotrópico (27% vs 4%, p = 0,002). No que tange aos exames laboratoriais, o grupo com comprometimento cardiovascular apresentou maior frequência de troponina elevada (33% vs 12%, p = 0,01), alanina transaminase elevada (33% vs 12%, p = 0,02) e de trombocitopenia (46% vs 22%, p = 0,02). A análise de curva ROC mostrou uma área sob a curva (AUC) de perfil laboratorial anormal (troponina elevada, alanina transaminase elevada, ou trombocitopenia) de 0.75, com 94% de sensibilidade e 98% de valor preditivo negativo para identificar necessidade de admissão à unidade de terapia intensiva.^9^

Em um grupo de 33 pacientes pediátricos críticos com COVID-19 admitidos em centro único no Brasil, Kozak et al. detectaram maior frequência de elevação da troponina em pacientes com SIM-P do que em pacientes sem SIM-P (77,8% vs 20.8%; p = 0,002). Além disso, o valor preditivo negativo da troponina elevada à admissão para a identificação de apcientes com ecocardiogramas alterados foi de 100% no grupo com SIM-P e de 73,7% no grupo sem SIM-P. Os autores sugeriram que o nível de troponina à admissão pode ser um parâmetro valioso para a identificação dos pacientes com necessidade de realização de um ecocardiograma em caráter de urgência, em um sistema público de saúde já sobrecarregado.^10^

Concluíndo, estudos maiores devem ser conduzidos em cenários de maior vulnerabilidade socioeconômica, com o intuito de mapear ferramentas amplamente disponíveis e de baixo custo para o diagnóstico de comprometimento cardiovascular de pacientes pediátricos com COVID-19 no momento de sua admissão. Isso é de vital importância para a decisão clínica, frente a recursos limitados de cuidados intensivos.
