# Supplementary material for: Como Devemos Investigar a Lesão Cardiovascular em Pacientes Pediátricos com COVID-19 Criticamente Enfermos em um Cenário de Vulnerabilidade Socioeconômica?
Source: Arq Bras Cardiol. 2022 May 4;118(5):946–8. [Article in Portuguese] doi: 10.36660/abc.20220254 (PMC9368879; doi:10.36660/abc.20220254)
Supplement: Translation

# Translation: How Should We Investigate Cardiovascular Injury In Critically Ill COVID-19 Pediatric Patients In A Scenario Of Socioeconomic Vulnerability?

**Authors:** Gabriela Nunes Leal

**Affiliations:** 1 Brasil Instituto da Criança e do Adolescente do Hospital das Clínicas da Faculdade de Medicina da Universidade de São Paulo, São Paulo, SP - Brasil; 2 Brasil Hospital Sírio Libanês, São Paulo, SP - Brasil; 3 Brasil Hospital do Coração, São Paulo, SP - Brasil; 4 Brasil Hospital e Maternidade São Luiz Itaim, São Paulo, SP - Brasil

**Keywords:** Children, COVID-19/complications, Cardiovascular Diseases/complications, Severe Acute Respiratory Syndrome, Inflammation, Hospitalization

Literature published worldwide has extensively documented cardiovascular injury among COVID-19 critically ill patients. Cardiac involvement appears to be a prominent feature of the disease in adults, occurring in 20% to 30% of hospitalized patients and contributing to 40% of deaths.^1^ Children and adolescents are mostly spared by COVID-19, with few having severe symptoms and even fewer deaths. However, the description of the multisystem inflammatory syndrome in children (MIS-c) reinforced that, although rare, severe clinical presentation and death are possible in the pediatric population.^2^ Cardiovascular compromise in MIS-c associated with COVID-19 is frequent and is one of the World Health Organization (WHO) diagnostic criteria for this pathologic condition ( Figure 1 ).^3 , 4^

**Figure 1:** – World Health Organization (WHO) definition criteria for multisystem inflammatory syndrome in children (MIS-c), associated with COVID-19.

Feldstein et al. detected 80% of cardiac compromise in a group of 186 MIS-c patients from 26 American states. Of note, 91% of these patients had at least one echocardiogram performed during their hospital stay.^5^ Recent national data documented 48% of echocardiographic abnormalities in a single-center cohort of hospitalized COVID-19 pediatric patients, associated with MIS-c, admission to the pediatric intensive care unit, multiple organ dysfunction, the need for ventilatory/vasoactive support, and death. In the same study, ventricular systolic dysfunction and coronary artery aneurysms detected by echocardiogram were associated with higher levels of troponin and d-dimer and inflammatory biomarkers.^6^

Due to COVID-19 in children, higher mortality rates have been recorded in Brazil compared with other countries (8.2% x 1%), mainly due to socioeconomic vulnerability and poor access to appropriate medical support. Since data on out-of-Hospital mortality is frequently missing, underestimating the pandemic effect on our pediatric population is expected.^7^

According to robust data collected from 5857 patients younger than 20 years old, all hospitalized with laboratory-confirmed COVID-19, ethnic, regional and socioeconomic conditions seem to shape the mortality of children with COVID-19 in Brazil. Compared with white children, indigenous and mixed-race children had significantly higher odds of mortality (OR 5.83, 95% CI 2.43 to 14.02; OR 1.93, 95% CI 1.48 to 2.51, respectively). The authors also found a regional influence (higher mortality in the North - OR 3.4, 95% CI 2.48 to 4.65) and a socioeconomic association (lower mortality among children from more socioeconomically developed municipalities - OR 0.26, 95% CI 0.17 to 0.38).^8^

Life-threatening cardiovascular complications in a resource-poor setting may be unrecognized, contributing to unfavorable outcomes in critically ill COVID-19 pediatric patients. High-cost imaging tools, such as Computed Tomography or Cardiac Magnetic Resonance, are usually unavailable. Even a bedside echocardiogram, suggested by most current MIS-c management guidelines, may not be accessible at admission.^9^

Identifying laboratory parameters at early presentation may trigger heightened suspicion of cardiovascular impairment and the need for intensive care management, particularly in the absence of imaging resources.

A multinational study of LATAM youth examined the distinguishing features of severe acute respiratory syndrome due to SARS-COV 2 infection and MIS-c, with versus without cardiac involvement. Ninety-eight patients from 32 centers in 10 countries of Central America, South America, and Mexico were included. The cardiac group was defined as diagnosed with arrhythmia, including premature atrial or ventricular contractions, sustained or non-sustained atrial or ventricular tachycardias, or atrioventricular block of any degree; dilation of any coronary artery segment (z-score > +2); left ventricle ejection fraction below 50%; dilated left ventricle (diastolic diameter z-score > +2); qualitative assessment of moderate or greater regurgitation of atrioventricular or semilunar valves; any pericardial effusion; clinical diagnosis of myocarditis by treating provider; clinical diagnosis of peripheral edema; or vascular thrombus/embolism.^9^ Forty-eight patients showed cardiac involvement, and 50 did not. The cardiac group had higher frequency of ICU admission (77% vs 54%, p = 0.02); invasive ventilation (23% vs 4%, p = 0.007) and vasoactive support (27% vs 4%, p = 0.002). Regarding laboratory profile, cardiac group had higher frequency of elevated troponin (33% vs 12%, p = 0.01), elevated alanine aminotransferase (33% vs 12%, p = 0.02) and thrombocytopenia (46% vs 22%, p = 0.02). Receiver operating curve analysis showed that abnormal laboratory profile (elevated troponin, elevated alanine aminotransferase or thrombocytopenia) had an area under the curve of 0.75, with 94% sensitivity and 98% negative predictive value on the need for intensive care unit.^9^

In a group of 33 critically ill COVID-19 pediatric patients admitted to a single center in Brazil, Kozak *et al.* detected a higher frequency of troponin elevation in MIS-c patients than in non-MIS-c patients (77.8% vs. 20.8%; p = 0.002). Moreover, the negative predictive value of elevated troponin at admission for detecting echocardiographic abnormalities was 100% in the MIS-C group and 73.7% in the non-MIS-c group. The authors suggest that troponin level at admission may be a valuable parameter to identify patients in more urgent need of an echocardiogram in an overwhelmed public health system.^10^

In conclusion, larger studies must be held in vulnerable socioeconomic scenarios to identify low-cost and widely available tools for detecting COVID-19 pediatric patients with cardiovascular involvement at admission. This is of uttermost importance for clinical decision-making in the face of limited pediatric intensive care unit resources.
